# Serum total TDP-43 levels are decreased in frontotemporal dementia patients with *C9orf72* repeat expansion or concomitant motoneuron disease phenotype

**DOI:** 10.1186/s13195-022-01091-8

**Published:** 2022-10-11

**Authors:** Kasper Katisko, Nadine Huber, Tarja Kokkola, Päivi Hartikainen, Johanna Krüger, Anna-Leena Heikkinen, Veera Paananen, Ville Leinonen, Ville E. Korhonen, Seppo Helisalmi, Sanna-Kaisa Herukka, Valentina Cantoni, Yasmine Gadola, Silvana Archetti, Anne M. Remes, Annakaisa Haapasalo, Barbara Borroni, Eino Solje

**Affiliations:** 1 Institute of Clinical Medicine – Neurology, University of Eastern Finland, P.O. Box 1627 (Yliopistonranta 1C), FI-70211 Kuopio, Finland; 2 A.I. Virtanen Institute for Molecular Sciences, University of Eastern Finland, Kuopio, Finland; 3 Neuro center, Neurology, Kuopio University Hospital, Kuopio, Finland; 4 Research Unit of Clinical Neuroscience, Neurology, University of Oulu, Oulu, Finland; 5 MRC, Oulu University Hospital, Oulu, Finland; 6 Neurology, Neurocenter, Oulu University Hospital, Oulu, Finland; 7 Finnish Institute of Occupational Health, Work Ability and Working Careers, Helsinki, Finland; 8 Neuro Center, Neurosurgery, Kuopio University Hospital, 70029 Kuopio, Finland; 9 Institute of Clinical Medicine – Neurosurgery, University of Eastern Finland, 70211 Kuopio, Finland; 10 Institute of Clinical Medicine, Internal Medicine, University of Eastern Finland, Kuopio, Finland; 11 Centre for Neurodegenerative Disorders, Department of Clinical and Experimental Sciences, University of Brescia, Brescia, Italy; 12 ASST Spedali Civili, Brescia, Italy; 13 Unit of Clinical Neuroscience, Neurology, University of Oulu, Oulu, Finland; 14 Medical Research Center, Oulu University Hospital, Oulu, Finland

**Keywords:** Frontotemporal dementia, Frontotemporal lobar degeneration, TDP-43 proteinopathy, C9orf72, GRN, Diagnostics, Disease progression, Biomarker

## Abstract

**Background:**

Frontotemporal dementia (FTD) covers a spectrum of neurodegenerative disorders with various clinical and neuropathological subtypes. The two major pathological proteins accumulating in the brains of FTD patients, depending on their genetic background, are TDP-43 and tau. We aimed to evaluate whether total TDP-43 levels measured from the serum associate with the genotype or clinical phenotype of the FTD patients and whether serum TDP-43 provides prognostic or diagnostic value in the FTD spectrum disorders.

**Methods:**

The study cohort included 254 participants with a clinical diagnosis of FTD (including all major genotypes and clinical phenotypes) and 105 cognitively healthy controls. Serum total TDP-43 levels measured with a single-molecule array (Simoa) were compared within the FTD group according to the genotype, clinical phenotype, and predicted neuropathological subtype of the patients. We also evaluated the associations between the TDP-43 levels and disease severity or survival in FTD.

**Results:**

Total TDP-43 levels in the serum were significantly lower in the FTD group as compared to the healthy control group (275.3 pg/mL vs. 361.8 pg/mL, *B* = 0.181, 95%CI = 0.014–0.348, *p* = 0.034). The lowest TDP-43 levels were observed in the subgroup of FTD patients harboring predicted TDP-43 brain pathology (FTD-TDP, 241.4 pg/mL). The low levels in the FTD-TDP group were especially driven by *C9orf72* repeat expansion carriers (169.2 pg/mL) and FTD patients with concomitant motoneuron disease (FTD-MND, 113.3 pg/mL), whereas *GRN* mutation carriers did not show decreased TDP-43 levels (328.6 pg/mL). Serum TDP-43 levels showed no correlation with disease severity nor progression in FTD.

**Conclusions:**

Our results indicate that the total levels of TDP-43 in the serum are decreased especially in FTD patients with the *C9orf72* repeat expansion or FTD-MND phenotype, both subtypes strongly associated with TDP-43 type B brain pathology. Serum-based measurement of TDP-43 could represent a useful tool in indicating *C9orf72* repeat expansion and FTD-MND-related TDP-43 neuropathology for future diagnostics and intervention studies.

## Background

Frontotemporal dementia (FTD) spectrum comprises a group of progressive neurodegenerative disorders presenting variable clinical, genetic, and neuropathological subtypes. The clinical subtypes include behavioral variant frontotemporal dementia (bvFTD), primary progressive aphasias (PPA) including the non-fluent variant (nfvPPA) or the semantic variant (svPPA), FTD-plus phenotypes of FTD as corticobasal degeneration (CBD) or progressive supranuclear palsy (PSP), and FTD with concomitant motoneuron disease (FTD-MND) [1, 2]. The most prevalent genetic causes of FTD are the hexanucleotide repeat expansion in *C9orf72* (*C9-*HRE) and mutations in the *GRN* or *MAPT* genes [3]. Neuropathologically, the brains of FTD patients are characterized by the presence of intracellular protein inclusions, which most typically contain either TAR DNA-binding protein 43 (TDP-43) or tau protein. Previous studies have shown that TDP-43 neuropathology associates with the *C9-*HRE and *GRN* mutations, whereas the brain pathology of *MAPT* mutation carriers is characterized by tau-positive inclusions [1, 2]. Of the clinical phenotypes, FTD-MND is strongly associated especially with TDP-43 pathology, PSP and CBD with tau pathology, and bvFTD and PPA with both pathologies [4].

The definite diagnosis of FTD remains a diagnostic challenge due to the clinical overlap with other neurodegenerative diseases and primary psychiatric disorders (PPD), especially in the early phases of the disease, as well as due to the heterogeneous nature of the underlying FTD neuropathology. At present, the definite FTD diagnosis requires either detection of a causal genetic mutation or neuropathological confirmation in the post-mortem examination. Identification of specific biomarkers, which would reflect the neuropathological subtype already at the early diagnostic phase in the patients, is crucial for improving the early diagnostics of FTD and conducting successful therapeutic trials in the future. Present ultrasensitive analytical methods, such as single-molecule array (Simoa), have enabled measurements of central nervous system-derived proteins, which may be present at low abundance, from the cerebrospinal fluid (CSF) but also from peripheral blood samples. The latter are more easily and widely accessible as well as less invasive, and thus, their sampling may be more practical, e.g., in clinical settings. Our prior studies, together with those from others, have shown that neurofilament light chain (sNfL) and glial fibrillary acidic protein (sGFAP) can be reliably detected from the serum samples of FTD patients [5–10]. Furthermore, our examinations suggested that sNfL and sGFAP measurements alone or in combination may provide an easily accessible, minimally invasive, and sensitive tool for the differential diagnostics between FTD and PPD patients and a prognostic tool predicting survival and brain atrophy rate in FTD patients [5–7]. However, it should be noted that neither NfL nor GFAP are specific markers for FTD patients only but rather represent indicators of neurodegeneration and glial cell activation or neuroinflammation generally in different neurodegenerative diseases [9, 11]. Therefore, other specific biomarkers, including those which could predict brain pathology already during the patients’ lifetime, are needed for a more comprehensive diagnostic subtyping of the patients within the heterogeneous FTD spectrum.

Development of a reliable biomarker indicating TDP-43 pathology is challenging as the TDP-43 protein itself is ubiquitously expressed (present in both central and peripheral tissues) and as different assay antibodies may have different binding capabilities towards different forms of the TDP-43 protein (i.e., physiological and pathological forms). In a few previous studies, total TDP-43 or phosphorylated TDP-43 (pTDP-43) levels have been measured from blood or CSF samples of patients with FTD/ALS spectrum disorders using standard ELISA-based methods. Higher levels of pTDP-43 especially in the patients with suspected TDP-43 pathology (including patients with MND or FTD-MND phenotype or patients with TDP-43 pathology-associated mutations) have been reported [12]. Interestingly, the levels of total TDP-43 in the blood and CSF have been shown to inversely correlate with those of pTDP-43, indicating lower total TDP-43 levels in the FTD-TDP groups [13, 14]. However, fairly small sample sizes have been used in previous studies in genetic FTD cases [14]. Therefore, they have suffered from a limited performance of the less sensitive ELISA methods, which have been unable to detect TDP-43 proteins. Due to this, a significant proportion of cases have been excluded from those studies. Moreover, contradicting results have been reported regarding the differences related to pTDP-43 and total TDP-43 levels in ALS/FTD patients, as, for instance, elevated levels of both total and pTDP-43 have been observed in ALS patients [12, 15].

Here, we have used the ultrasensitive Simoa method to measure the levels of serum total TDP-43 in a large multicenter cohort of FTD patients. We compared the TDP-43 levels within the FTD group between all major genotypes and clinical phenotypes and further evaluated whether the TDP-43 levels associated with disease severity or progression rate of the FTD patients. Furthermore, we compared the serum TDP-43 levels between FTD patients and cognitively healthy controls.

## Methods

### Patients and controls

Altogether, 254 FTD patients were included in the study of whom 107 were recruited at the memory outpatient clinic of the Department of Neurology, Kuopio University Hospital (Finland), and 147 at the Centre for Neurodegenerative Disorders, University of Brescia (Italy). The eventual clinical phenotypes of the 254 FTD patients were as follows: 125 bvFTD, 63 nfvPPA, 33 svPPA, 24 PSP, and 9 FTD-MND (Table 1). Patients were diagnosed according to the most recent clinical criteria [16–18] by neurologists specialized in neurodegenerative disorders, including neurological examination for concomitant signs of motoneuron disease. Patients with FTD-MND met the criteria for either bvFTD or PPA and also at least clinically possible ALS according to El Escorial criteria [19]. FTD patients diagnosed before 2011 were originally diagnosed according to the Neary 1998 criteria [20] and retrospectively evaluated to meet the most recent diagnostic criteria. All FTD patients met at least the probable criteria for bvFTD, PPA, PSP, or FTD-MND. In total, 61 patients received a definite FTD diagnosis due to genetic (60) or neuropathological (1) confirmation. Of the FTD-associated genetic mutations, there were 26 *C9-*HRE carriers, 31 *GRN* mutation carriers, and 3 *MAPT* mutation carriers. Disease severity of the FTD patients was evaluated with Frontotemporal Dementia Clinical Dementia Rating (FTD-CDR) sum of boxes score (Table 1).

**Table 1:** Demographics of the study cohort with serum TDP-43 levels in each group

Clinical phenotype/genetic background	FTD total, *N* = 254	FTD-TDP, *N* = 64	FTD-tau, *N* = 27	HC, *N* = 105
bvFTD (*N*)	125	37	3	–
*C9*-HRE (*N*)	18	18	0	–
*GRN* (*N*)	18	18	0	–
*MAPT* (*N*)	2	0	2	–
nfvPPA (*N*)	63	18	0	–
*C9*-HRE (*N*)	5	5	0	–
*GRN* (*N*)	13	13	0	–
*MAPT* (*N*)	0	0	0	–
svPPA (*N*)	33	0	0	–
*C9*-HRE (*N*)	0	0	0	–
*GRN* (*N*)	0	0	0	–
*MAPT* (*N*)	0	0	0	–
PSP (*N*)	24	0	24	–
*C9*-HRE (*N*)	0	0	0	–
*GRN* (*N*)	0	0	0	–
*MAPT* (*N*)	1	0	1	–
FTD-MND (*N*)	9	9	0	–
*C9*-HRE (*N*)	3	3	0	–
*GRN* (*N*)	0	0	0	–
*MAPT* (*N*)	0	0	0	–
Gender, female %	52%	52%	44%	56%
Age, years, mean (SD)	66.1 (9.0)	62.2 (9.2)	69.0 (10.5)	67.2 (9.8)
FTD-CDR Sum of Boxes, mean (SD)	6.2 (4.4)	7.5 (4.7)	4.2 (3.0)	–
Serum TDP-43, pg/mL, median (interquartile range, IQR)	204.8* (247.8)	190.3^#,^^ (222.6)	305.8^^^ (290.3)	253.1^*,#^ (258.8)

*Abbreviations*: *bvFTD* behavioral variant frontotemporal dementia, *C9-HRE C9orf72* hexanucleotide repeat expansion, *FTD* frontotemporal dementia, *FTD-CDR* Frontotemporal dementia Clinical Dementia Rating score, *FTD-MND* frontotemporal lobar degeneration with motoneuron disease, *FTD-tau* frontotemporal dementia with tau pathology, *FTD-TDP* frontotemporal dementia with TAR DNA-binding protein pathology, *HC* healthy controls, *nfvPPA* non-fluent variant of primary progressive aphasia, *PSP* progressive supranuclear palsy, *SD* standard deviation, *svPPA* semantic variant of primary progressive aphasia, *TDP-43* TAR DNA-binding protein 43

Significant *p*-values in TDP-43 level comparisons adjusted for age and sex: *p = 0.034, FTD total vs. HC; ^#^*p* = 0.036, FTD-TDP vs. HC; ^^^*p* = 0.022, FTD-TDP vs. FTD-tau

To allow comparisons according to the predicted neuropathological status, subgroups of FTD-TDP and FTD-tau were generated by including the *C9-*HRE carriers, *GRN* mutation carriers, and patients with the FTD-MND phenotype to the FTD-TDP group (*N* = 64) and the *MAPT* mutation carriers and patients with the PSP phenotype to the FTD-tau group (*N* = 27).

In comparisons according to the genotypes, the *C9-*HRE carrier group, the *GRN* mutation carrier group, and the *MAPT* mutation carrier group were compared to the non-genetic FTD group (*N* = 170). FTD patients in the non-genetic group did not carry the *C9-*HRE, and the *MAPT* and *GRN* mutations were excluded from the non-genetic Italian patients. *MAPT* or *GRN* mutations were not systematically tested in non-genetic Finnish FTD patients, but our previous genetic studies have unequivocally indicated that these mutations are extremely rare or non-existent in Finnish FTD patients [21–23]. Finnish FTD patients for whom confirmed genetic information regarding the *C9-*HRE carriership was not available (*N* = 25) were excluded from the analyses comparing the different genetic subgroups.

The healthy control (HC) group included 105 participants who completed cognitive testing and were assessed by a neuropsychologist and a neurologist and confirmed not to have a neurodegenerative disorder. Of the HC group, 19 were recruited from Oulu University Hospital Finland’s memory department. These were individuals with subjective memory complaints, who showed no signs of cognitive disorder or impairment after baseline neurological and neuropsychological evaluations, MRI, and follow-up visits after 6 and 12 months. From Kuopio University Hospital Finland, 86 participants were recruited among patients scheduled for knee replacement surgery or patients requiring spinal anesthesia for other reasons. Cognitive impairment was excluded with neuropsychological and neurological evaluation at baseline, and none of the participants had developed neurodegenerative disorder after 3 years of follow-up.

### Simoa analysis of total TDP-43 levels in serum samples

Total TDP-43 levels were quantified from a total of 359 serum samples according to the manufacturer’s instructions using the digital immunoassay technology on a single-molecule array (Simoa) HD-1 Analyzer, software version 1.5.1809.12001, and human TDP-43 Advantage Kit (REF# 103293) [Quanterix, Billerica, MA, USA] [24]. The Simoa TDP-43 assay has been developed with antibodies against amino acids 203–209 and the C-terminal region. According to the manufacturer, the assay is expected to detect both full-length and pathological truncated forms of TDP-43 protein. All samples were collected in the morning, rapidly divided into aliquots, and stored at − 80 °C. The samples were handled without repeated freeze-thawing. Before measurements, the samples were thawed at room temperature, gently mixed, and centrifuged (10,000*×g*, 5 min, + 20 °C). Samples were randomized on plates and measured in duplicates. A maximum coefficient of variation (CV) of sample replicates of 20% was accepted. The concentrations of all samples were above the lower limit of quantification (8.23 pg/mL) and within the dynamic range (0–8000 pg/mL) of the assay.

### Statistical analyses

Statistical analyses were performed using IBM SPSS Statistic version 25 and GraphPad Prism Software version 5 and version 9. The chi-square analysis was used to compare gender distribution and independent sample *t*-test (or Mann-Whitney *U* for non-parametric variables) to compare age and neuropsychological data distribution between the groups. Correlations between serum TDP-43 levels and other continuous variables were analyzed with Spearman’s rank correlation test or Pearson’s correlation test (depending on the variable distribution). Due to the non-normally distributed TDP-43 data (evaluated by the Shapiro-Wilk test and visual inspection), natural logarithmic transformation was performed (ln-sTDP-43). Differences in serum TDP-43 levels between the diagnostic groups and between the genetic and predicted neuropathological subgroups were evaluated with a multivariate linear regression model adjusted for age and sex. When evaluating diagnostic accuracy, receiver operating characteristics (ROC) curve analysis was used to calculate the area under the curve (AUC) values (Youden index method for cutoff evaluation). A *p*-value ≤ 0.05 was considered as statistically significant.

## Results

Demographics of the study cohort are shown in Table 1.

Serum TDP-43 levels showed no significant correlation with age at baseline in the total study cohort (FTD + HC, *N* = 359; *r* = 0.095, *p* = 0.075) or separately in the FTD group (*N* = 254, *r* = 0.016, *p* = 0.807) but showed a significant correlation in the HC group only (*N* = 105, *r* = 0.239, *p* = 0.014). When the effect of gender was considered, males had a trend for slightly higher levels of serum TDP-43, but the age-adjusted gender differences were non-significant in the total study cohort (*N* = 359, *p* = 0.052), in the HC group (*N* = 105, *p* = 0.072), and in the FTD group (*N* = 254, *p* = 0.285).

Analysis of the serum TDP-43 levels in the total group of the FTD patients (*N* = 254) vs. the HC group (*N* = 105) indicated that the levels were significantly lower in the FTD patients compared to HC (275.3 pg/mL vs. 361.8 pg/mL, *B* = 0.181, 95%CI = 0.014–0.348, *p* = 0.034) when adjusted for age and gender (Table 1).

**Fig. 1:**
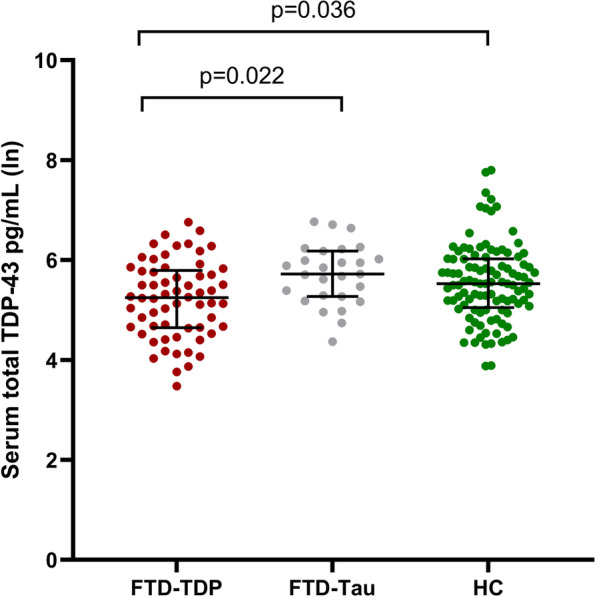
Serum total TDP-43 concentrations in FTD-TDP patients, FTD-tau patients, and healthy controls (HC). The values were obtained using natural logarithmic transformation. Black horizontal lines indicate the group median and interquartile ranges. *p*-values are calculated with a general linear model with age and gender as covariates. Only significant *p*-values are indicated

Furthermore, serum TDP-43 levels were significantly lower in the FTD-TDP group (*N* = 64) compared to the HC group (*N* = 105) (241.4 pg/mL vs. 361.8 pg/mL, *B* = 0.268, 95%CI = 0.018–0.517, *p* = 0.036) after similar adjustments (age and gender), whereas the TDP-43 levels did not significantly differ between the FTD-tau group (*N* = 27) and the HC group (*N* = 105) (356.9 pg/mL vs. 361.8 pg/mL, *B* = − 0.092, 95%CI = − 0.400–0.215, *p* = 0.554) (Table 1, Fig. 1).

Between the predicted neuropathological subgroups, FTD-TDP group (*N* = 64) showed lower serum TDP-43 levels compared to the FTD-tau group (*N* = 27) (241.4 pg/mL vs. 356.9 pg/mL, *B* = 0.416, 95%CI 0.061–0.771, *p* = 0.022) when adjusted for age and gender (Table 1, Fig. 1).

**Fig. 2:**
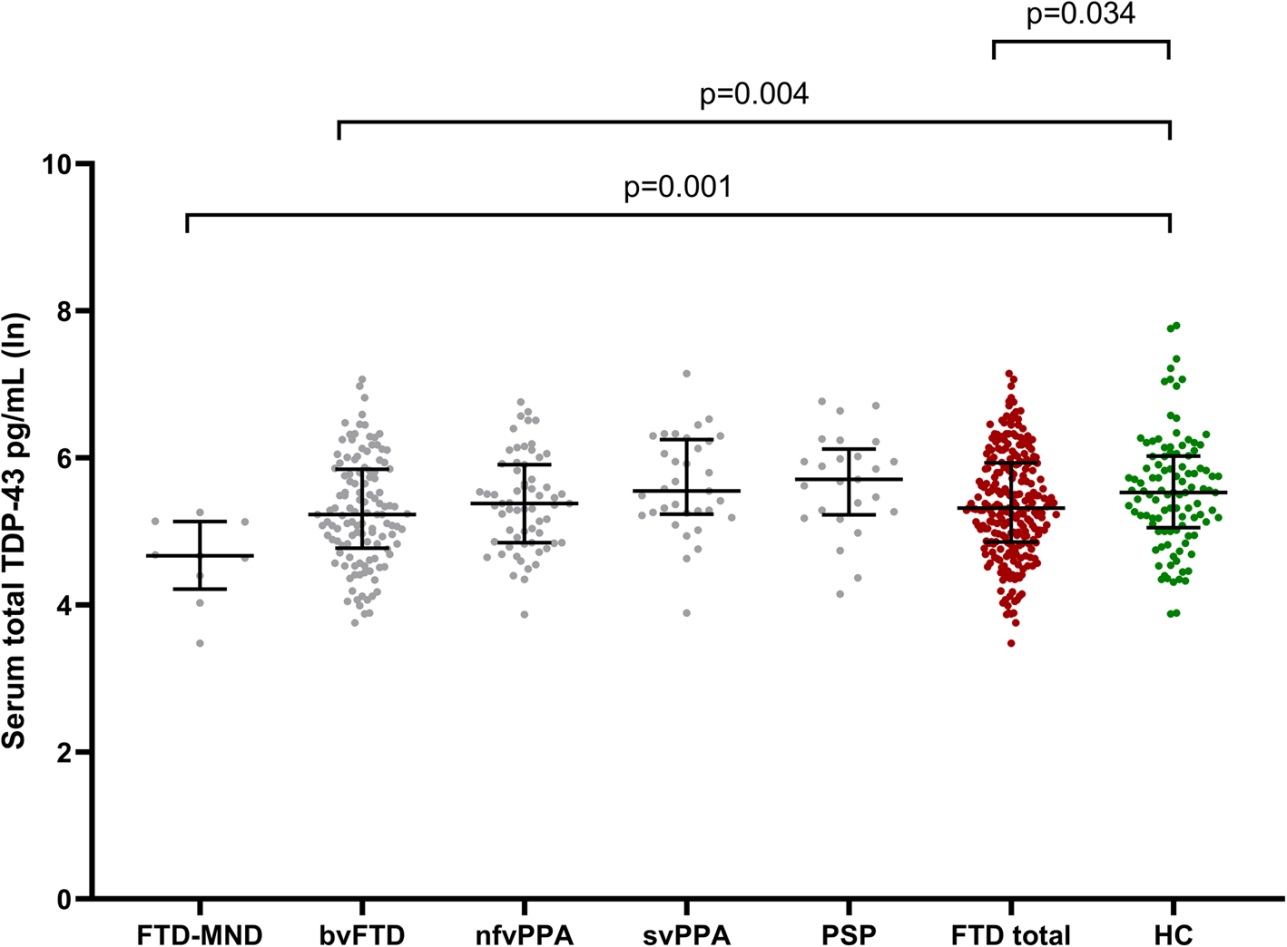
Serum total TDP-43 concentrations in individual subgroups based on the clinical phenotypes of frontotemporal dementia (FTD), in the total FTD group including all of the phenotypes, and in healthy controls (HC). The values were obtained using natural logarithmic transformation. Black horizontal lines indicate group median and interquartile ranges. *p*-values are calculated with a general linear model with age and gender as covariates. Only significant *p*-values are indicated. *Abbreviations*: TDP-43, TAR DNA-binding protein 43; FTD-MND, frontotemporal dementia with motor neuron diseases; bvFTD, behavioral variant frontotemporal dementia; nfvPPA, non-fluent variant primary progressive aphasia; svPPA, semantic variant primary progressive aphasia; PSP, progressive supranuclear palsy

Next, we analyzed the serum TDP-43 levels in patients with different clinical phenotypes of FTD. This analysis revealed that the lowest levels of serum TDP-43 were present in the patients with the FTD-MND phenotype (*N* = 9, 113.3 pg/mL) (i.e., TDP43-positive cases), while the highest levels were found in PSP patients (*N* = 24, 362.6 pg/mL) (i.e., tau-positive cases). The levels in bvFTD patients (*N* = 125, 254.1 pg/mL), nfvPPA patients (*N* = 63, 269.4 pg/mL), and svPPA patients (*N* = 33, 344.9 pg/mL) were in between these extremes. When compared to the HC group (*N* = 105, 361.8 pg/mL) adjusted for age and sex, statistically significant differences were observed between FTD-MND and HC (*B* = 0.859, 95%CI = 0.314–1.376, *p* = 0.001) and between bvFTD and HC (*B* = 0.288, 95%CI = 0.090–0.486, *p* = 0.004) (Fig. 2).

**Fig. 3:**
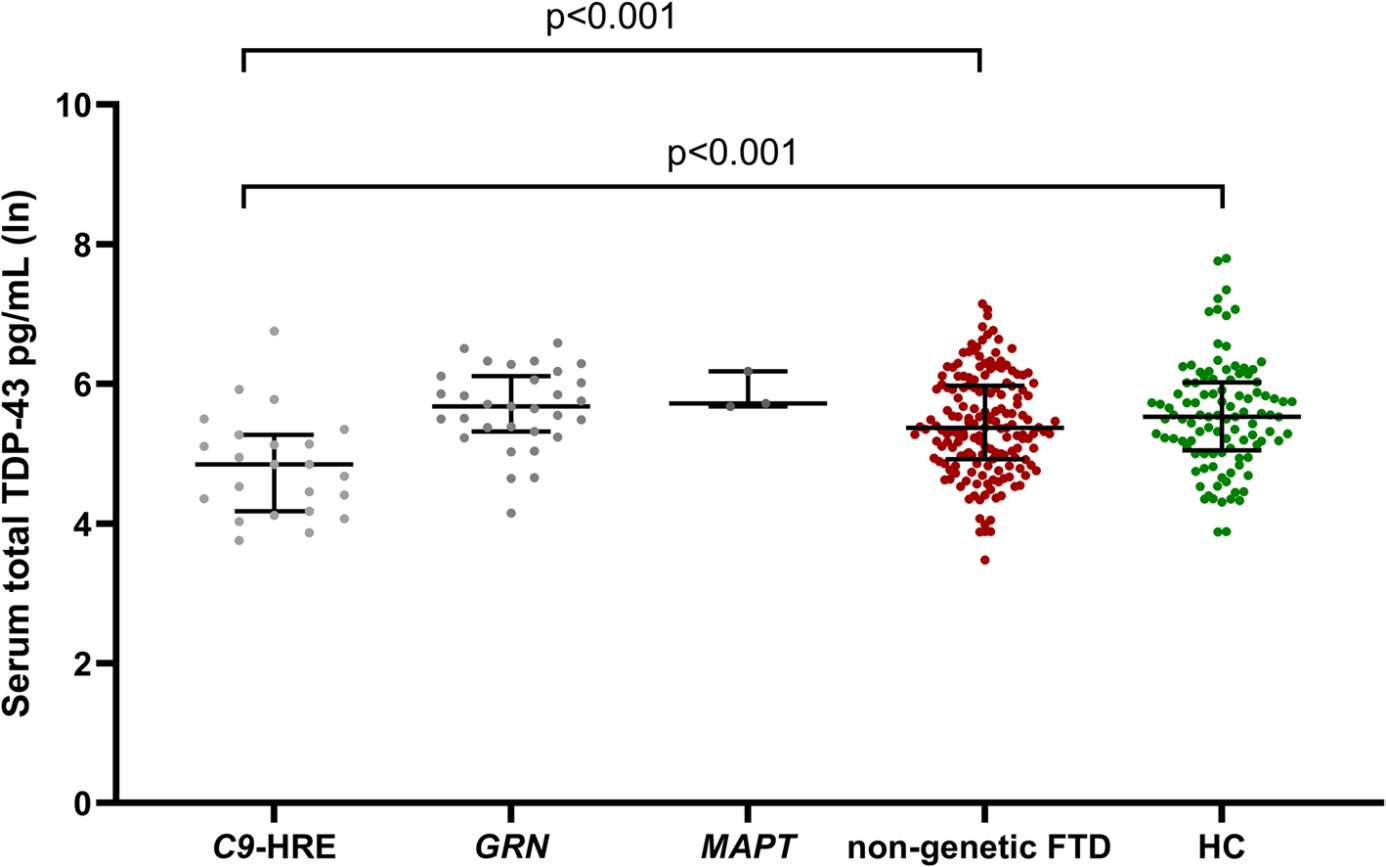
Serum total TDP-43 concentrations in the different genetic groups of frontotemporal dementia (FTD), in non-genetic FTD patients, and in healthy controls (HC). The values were obtained using natural logarithmic transformation. Black horizontal lines indicate group median and interquartile ranges. *p*-values are calculated with a general linear model with age and gender as covariates. Only significant *p*-values are indicated. *Abbreviations*: TDP-43, TAR DNA-binding protein 43; *C9-*HRE, Chromosome 9 open reading frame 72 hexanucleotide repeat expansion; *GRN*, progranulin mutation; *MAPT*, microtubule-associated protein tau mutation

When compared to the HC group (361.8 pg/mL), a statistically significant difference was observed between *C9-*HRE carriers (*n* = 26) and HC (*N* = 105) (169.2 pg/mL, *B* = 0.634, 95%CI = 0.284–0.984, *p* < 0.001), while no significant differences were found between the *GRN* mutation carriers (*N* = 31) (328.6 pg/mL) vs. HC (*B* = − 0.180, 95%CI = − 0.473–0.113, *p* = 0.226). Furthermore, as expected, no differences between *MAPT* mutation carriers (*N* = 3) (361.6 pg/mL) and HC (361.8 pg/mL) (not compared statistically due to limited group size of the *MAPT* mutation carriers) or non-genetic FTD (*N* = 170) (291.9 pg/mL) vs. HC (*B* = 0.138, 95%CI = − 0.041–0.317, *p* = 0.130) (adjusted for age and sex) were detected. When the genetic FTD patient groups (*C9-*HRE, *GRN*, or *MAPT*) were independently compared to the non-genetic FTD group, only the difference between *C9*-HRE and non-genetic FTD was statistically significant (*B* = 0.604, 95%CI = 0.281–0.926, *p* < 0.001) (Fig. 3).

In receiver operating curve (ROC) analyses, the best diagnostic accuracies of serum TDP-43 levels were observed between *C9*-HRE carriers and HC (area under the curve (AUC) = 0.765, with 72% sensitivity and 75% specificity, cutoff 174 pg/mL) and between FTD-MND and HC (AUC = 0.848, with 72% sensitivity and 89% specificity, cutoff 174 pg/mL). In the ROC analysis between the FTD-TDP and FTD-tau groups, the AUC was 0.689 with 63% sensitivity and 68% specificity (cutoff 267 pg/mL). *C9*-HRE and the FTD-MND phenotype are associated with TDP type B pathology, whereas *GRN* mutations are associated with TDP type A pathology [25]. When *GRN* mutation carriers were excluded from the whole FTD-TDP group to generate a more specific “FTD-TDP type B” subgroup and compared to the FTD-tau group, the AUC was 0.860 with 85% sensitivity and 77% specificity (cutoff 173 pg/mL).

Finally, we investigated if serum TDP-43 levels showed a correlation with the severity or progression of the disease in FTD patients. The results indicated that the serum TDP-43 levels did not correlate with disease severity when assessed by using the FTD-CDR sum of boxes score (*r*_s_ = 0.017, *p* = 0.807). The survival time was available for Finnish FTD patients (*N* = 107). Of these, 47/107 died during the follow-up, and the mean disease duration was 4 years. The serum TDP-43 levels did not associate with survival time after the diagnosis in the total FTD group (*r*_s_ = − 0.126, *p* = 0.445), in the FTD-TDP subgroup (*r*_s_ = − 0.236, *p* = 0.437), nor in the *C9orf72* repeat expansion carrier group (*r*_s_ = − 0.364, *p* = 0.272).

## Discussion

In the present study, our objective was to assess whether serum total TDP-43 levels measured from FTD patients harboring different genotypes and clinical phenotypes could be utilized to aid in the differentiation of FTD neuropathological subtypes or in disease prediction. To date, there are no approved and officially validated biomarkers available that would indicate the development of TDP-43 pathology in the brains of the patients during their lifetime. Our findings show that serum total TDP-43 levels in the overall FTD group are significantly lower than those in the HC group. Importantly, especially *C9*-HRE carriers and FTD-MND patients, who characteristically display TDP-43 neuropathology, showed lower serum TDP-43 levels as compared to the HC group, whereas no difference was observed between the HC group and FTD-tau patients, implicating that these findings are specific and relevant.

TDP-43 is a highly conserved and ubiquitously expressed RNA- and DNA-binding protein that belongs to the heterogeneous nuclear ribonucleoprotein family, centrally involved in RNA-related metabolism [26, 27]. Under physiological conditions, TDP-43 predominantly localizes in the nucleus, but it can undergo shuttling between the nucleus and the cytoplasm. TDP-43 is involved in multiple cellular functions, such as regulation of RNA metabolism, mRNA transport, and stress granule formation. It also plays a pivotal role in the regulation of transcription and mRNA splicing [28]. TDP-43 also appears to be critical for the development of neuronal cells during the early developmental stages [27]. In neurodegenerative TDP-43 proteinopathies, such as FTD or ALS, TDP-43 accumulates in cytoplasmic aggregates, which are cytotoxic. Moreover, this leads to an apparent loss of nuclear TDP-43, which may result in disrupted RNA metabolism and other cellular disturbances [29]. The TDP-43-positive inclusions, consisting of abnormally aggregated, truncated, ubiquitinated, and hyperphosphorylated TDP-43, associate with neurodegeneration and are present in the brain but can also occur in the spinal cord of the patients [30, 31].

Our findings showing decreased, rather than increased, total TDP-43 levels in the serum of FTD patients with suspected TDP-43 pathology are in line with a previous study that assessed both total TDP-43 and pTDP-43 in a small cohort of genetic FTD patients, including ten *C9*-HRE carriers and five *GRN* mutation carriers [14]. The study found an inverse correlation between total TDP-43 and pTDP-43 levels as pTDP-43 levels were higher and total TDP-43 levels lower in these genetic FTD patients with suspected TDP-43 pathology as compared to healthy controls. The authors hypothesized that the *C9*-HRE or *GRN* mutations alter the balance between the phosphorylated and non-phosphorylated forms of TDP-43, promoting the former one and resulting in an imbalance in their relative amounts [14]. In our cohort with a larger data set, especially the *C9*-HRE group was associated with low total TDP-43 levels, which might support the previous hypothesis that in TDP-43-positive proteinopathies, the p-TDP-43/TDP-43 ratio could be imbalanced. However, as the Simoa assay used in this study did not utilize antibodies separately detecting different post-translationally modified forms of TDP-43, it is difficult to estimate whether some specific forms of TDP-43 would be more specifically or preferentially recognized by the assay. Therefore, it presently remains unclear if the decreased levels of serum TDP-43 in this study could be explained by an imbalanced p-TDP-43/TDP-43 ratio or some other underlying mechanism(s), e.g., related to differential release or degradation of different TDP-43 forms. Future improved assays, specifically targeting specific TDP-43 forms will help in clarifying the reason underpinning the observed decrease in the serum TDP-43 levels.

Notably, the lowered levels of total TDP-43 in the FTD-TDP group were strongly driven by the *C9*-HRE genotype and FTD-MND phenotype indicating that mainly *C9*-HRE and FTD-MND-related TDP-43 pathophysiology (TDP type B pathology in *C9*-HRE carriers and FTD-MND patients vs. TDP type A pathology in *GRN* mutation carriers) is reflected to the total TDP-43 serum levels measured with Simoa assay. Our findings are in contrast to a previous study, which reported decreased plasma TDP-43 levels also in the *GRN* mutation carriers [14]. The underlying reason for the different observations regarding the total TDP-43 levels in *GRN* mutation-carrying patients in the present study as compared to the previous one [14] could be the small sample size (5 *GRN* patients) in the previous study or the fact that different assay methods were used to detect TDP-43 (ELISA in the previous study vs. ultrasensitive Simoa in the present study).

Lower levels of especially total TDP-43 have also been observed in the lumbar CSF of FTD-TDP patients [13], a finding that is well in accordance with our present data on reduced serum TDP-43 levels in FTD patients having predicted TDP-43 pathology. Lower levels of total TDP-43 in the CSF have been associated with shorter survival of ALS patients [32], suggesting that total TDP-43 levels could predict disease progression rate. However, we did not find evidence for such prognostic value in the serum-based measurement of TDP-43 levels in our present cohort with FTD-TDP patients. Notably, a study evaluating both total plasma TDP-43 and plasma pTDP-43 reported a positive correlation especially between pTDP-43 levels and brain TDP-43 deposition [33]. This might lead to the assumption that *C9*-HRE as well as *GRN* mutation carriers have a more pronounced TDP-43 accumulation in their brain and concurrently increased release of pTDP-43 from the degenerating neurons, indicated by the higher pTDP-43 levels in the biofluid samples. Given the previous study reporting an inverse correlation of the levels of pTDP-43 and total TDP-43 [14], it is possible that our results indicating lower levels of total serum TDP-43 could reflect increased brain accumulation of TDP-43 at least in *C9*-HRE/FTD-ALS related disease. Thus, lower levels of TDP-43 in the biofluids, such as serum, could resemble the behavior of β-amyloid as a biomarker in Alzheimer’s disease (AD) patients. It has been reported that decreased β-amyloid levels in the CSF or blood are a hallmark of increased β-amyloid accumulation and aggregation in AD brain [34, 35]. Similarly to β-amyloid in AD [34], the total TDP-43 levels do not appear to represent a general marker of neurodegeneration, as we did not find any correlation between the levels of serum TDP-43 and disease severity or progression. It should be noted that also contradicting findings to ours have been reported at least in pure ALS patients in whom both total TDP-43 and pTDP-43 levels in the plasma were higher than in healthy controls [15]. On the other hand, it appears that plasma TDP-43 levels may provide sufficient diagnostic accuracy between ALS patients and controls [15], and this outcome is similar to the one in the present study in the patients with FTD-ALS phenotype.

The main strength of the present study is the large and well-characterized multicenter cohort, which includes genetic patients carrying any of the three major FTD-associated mutations in either *C9orf72*, *GRN*, or *MAPT* as well as patients displaying diverse clinical phenotypes. Moreover, in comparison with some other previous studies, which utilized standard ELISA-based detection of TDP-43, we used here the ultrasensitive Simoa method, which allows the detection of targets with a low abundance. The levels of TDP-43 in none of our cases remained below the detection limit, which has been a substantial issue in the previous studies using other analysis methods. The limitations include the fact that although the genetic subgroups were the largest reported thus far, they still are fairly small when considering optimal statistical power in the subgroup analyses (for example, disease severity or prognostic analyses in the genetic or neuropathological subgroups). Another limitation is that we did not have the option to correlate the serum levels of TDP-43 to the levels or accumulation of TDP-43 (or pTDP-43) in the brain of the same patients. These analyses could be done in the future in other cohorts, which contain both biofluid and brain samples from the same individuals. They would be helpful for testing the hypothesis of whether lowered serum levels of TDP-43 reflect a more pronounced brain pathology, similarly to β-amyloid in the AD patients. Furthermore, the measurement of serum TDP-43 in a longitudinal setting might also provide further insight into this idea. It should also be noted that the Simoa TDP-43 assay used here is not specific for the pathological truncated and phosphorylated forms of TDP-43 as it detects also the full-length TDP-43 protein. Although significant differences were found between the groups, the overlap in the TDP-43 levels between several groups was substantial (e.g., FTD-tau vs. FTD-TDP), thus limiting the discriminative power of total TDP-43 levels detected using the present Simoa assay. Moreover, TDP-43 is not specifically expressed only in the central nervous system but is expressed also diversely in peripheral tissues. This might also partially explain the overlap between the groups and the differences between different studies. In the future, the diagnostic potential of TDP-43 measurements could be improved by combining measurements of TDP-43 levels with those of other biomarkers, or by developing specific and sensitive assays against different post-translationally modified TDP-43 protein forms, especially the truncated and phosphorylated forms, which associate with pathological changes in the brain.

## Conclusions

Our results demonstrate significantly lower serum total TDP-43 levels especially in the FTD patients with *C9*-HRE and FTD-MND related TDP-43 pathology as compared to healthy controls or patients with suspected tau pathology. This result may aid in the much-needed discovery of reliable biomarkers, which would enable distinguishing the different neuropathological subtypes of the FTD spectrum already in the early disease phases in living patients. The discovery of non-invasive, easily accessible, and reliable biomarkers is an essential step for accurate and timely diagnosis and, ultimately, for the development of specific therapeutic strategies targeting the underlying pathological mechanisms in different subtypes of FTD.

## Data Availability

The datasets generated and analyzed during the present study are available from the corresponding author upon reasonable request.

## Abbreviations

AD: Alzheimer’s disease
AUC: Area under the curve
bvFTD: Behavioral variant of frontotemporal dementia
CBD: Corticobasal degeneration
CSF: Cerebrospinal fluid
CV: Coefficient of variation
*C9*-HRE: Hexanucleotide repeat expansion in *C9orf72* gene
FTD: Frontotemporal dementia
FTD-CDR: Frontotemporal dementia Clinical Dementia Rating
FTD-MND: Frontotemporal dementia with motoneuron disease
FTD-tau: Frontotemporal dementia patients with predicted tau pathology
FTD-TDP: Frontotemporal dementia patients with predicted TDP-43 pathology
*GRN*: Gene encoding progranulin protein
HC: Healthy controls
*MAPT*: Microtubule-associated protein tau (gene)
nfvPPA: Non-fluent variant of frontotemporal dementia
PPA: Primary progressive aphasia
PPD: Primary psychiatric disorders
PSP: Progressive supranuclear palsy
pTDP-43: Phosphorylated TAR DNA-binding protein 43
RNA: Ribonucleic acid
ROC: Receiver operating characteristics
SGFAP: Serum glial fibrillary acidic protein
Simoa: Single-molecule array
SNfL: Serum neurofilament light
SvPPA: Semantic variant of frontotemporal dementia
TDP-43: TAR DNA-binding protein 43
